# The Antimalarial Compound Atovaquone Inhibits Zika and Dengue Virus Infection by Blocking E Protein-Mediated Membrane Fusion

**DOI:** 10.3390/v12121475

**Published:** 2020-12-21

**Authors:** Mizuki Yamamoto, Takeshi Ichinohe, Aya Watanabe, Ayako Kobayashi, Rui Zhang, Jiping Song, Yasushi Kawaguchi, Zene Matsuda, Jun-ichiro Inoue

**Affiliations:** 1 Research Center for Asian Infectious Diseases, The Institute of Medical Science, The University of Tokyo, Tokyo 113-0033, Japan; mizuyama@ims.u-tokyo.ac.jp (M.Y.); aya_w73@yahoo.co.jp (A.W.); ayamail824@gmail.com (A.K.); ykawagu@ims.u-tokyo.ac.jp (Y.K.); 2 Division of Cellular and Molecular Biology, Department of Cancer Biology, The Institute of Medical Science, The University of Tokyo, Tokyo 113-0033, Japan; 3 Division of Viral Infection, Department of Infectious Disease Control, International Research Center for Infectious Diseases, The Institute of Medical Science, The University of Tokyo, Tokyo 113-0033, Japan; ichinohe@ims.u-tokyo.ac.jp; 4 Laboratory of Structural Virology and Immunology, Institute of Biophysics, Chinese Academy of Sciences, Beijing 100864, China; ruizhangdmn@outlook.com; 5 China-Japan Joint Laboratory of Molecular Immunology & Microbiology, Institute of Microbiology, Chinese Academy of Sciences, Beijing 100864, China; songjiping2016@hotmail.com; 6 Division of Molecular Virology, Department of Microbiology and Immunology, Institute of Medical Science, The University of Tokyo, Tokyo 113-0033, Japan; 7 Department of Microbiology and Infection, Kochi Medical School, Kochi University, Kochi 780-8072, Japan; 8 Senior Professor Office, The University of Tokyo, Tokyo 113-0033, Japan

**Keywords:** Zika virus, Dengue virus, antiviral drugs, high-throughput screening, cell-based membrane fusion assay

## Abstract

Flaviviruses bear class II fusion proteins as their envelope (E) proteins. Here, we describe the development of an in vitro quantitative mosquito-cell-based membrane-fusion assay for the E protein using dual split proteins (DSPs). The assay does not involve the use of live viruses and allows the analysis of a membrane-fusion step independent of other events in the viral lifecycle, such as endocytosis. The progress of membrane fusion can be monitored continuously by measuring the activities of *Renilla* luciferase derived from the reassociation of DSPs during cell fusion. We optimized the assay to screen an FDA-approved drug library for a potential membrane fusion inhibitor using the E protein of Zika virus. Screening results identified atovaquone, which was previously described as an antimalarial agent. Atovaquone potently blocked the in vitro Zika virus infection of mammalian cells with an IC_90_ of 2.1 µM. Furthermore, four distinct serotypes of dengue virus were also inhibited by atovaquone with IC_90_ values of 1.6–2.5 µM, which is a range below the average blood concentration of atovaquone after its oral administration in humans. These findings make atovaquone a likely candidate drug to treat illnesses caused by Zika as well as dengue viruses. Additionally, the DSP assay is useful to study the mechanism of membrane fusion in Flaviviruses.

## 1. Introduction

Many recent emerging and re-emerging viral infectious diseases are caused by enveloped viruses [1]. Enveloped viruses use their envelope proteins to attach to target host cells in order to facilitate the introduction of their genome into the host cells via membrane fusion between the viral envelope and host–cell membrane [2,3,4]. Membrane fusion is the first essential step in the replication of these viruses and represents a major therapeutic target [5].

Envelope proteins can be classified into three categories based on the structural features of the fusion-protein subunit that mediates the actual membrane-fusion step [6]. Although class I fusion proteins comprise mainly α helices, β sheets are the major components of class II fusion proteins, whereas class III fusion proteins include mixed features from class I and II fusion proteins. Viruses harboring class I fusion proteins include human pathogens, such as influenza virus, Ebola virus, human immunodeficiency virus type 1 (HIV-1), and members of the coronavirus (CoV) genus including Middle East Respiratory Syndrome CoV (MERS-CoV), severe acute respiratory syndrome-CoV (SARS-CoV), and SARS-CoV-2. Viruses harboring class II fusion proteins include members of the Flavivirus genus, and those harboring class III fusion proteins include rhabdoviruses, herpesviruses, and baculoviruses.

Flaviviruses include several groups of insect-borne agents that represent a significant threat to human health, such as dengue virus (DENV), West Nile virus (WNV), yellow fever virus (YFV), Japanese encephalitis virus (JEV), and tick-borne encephalitis virus (TBEV) [7,8,9,10,11]. Zika virus (ZIKV) was recently identified as a re-emerging virus capable of potentially causing microcephaly in fetuses infected in utero [12,13,14]. Although the number of people affected by flaviviruses worldwide is quite large, no safe and effective antiviral drug for flaviviruses has been developed to date. Therefore, the development of specific therapeutic agents or vaccines is urgently needed.

Since membrane fusion is an essential step in infection by enveloped viruses, inhibitors of membrane fusion are expected to exhibit potent antiviral effects. We previously developed a quantitative split reporter-protein-based high-throughput cell-cell fusion assay for the MERS-CoV and SARS-CoV-2 S protein, resulting in identification of the serine protease inhibitor nafamostat as a potent inhibitor of S protein-mediated membrane fusion [15,16]. This was accomplished using the dual split reporter proteins (DSP)1-7 and DSP8-11, which represent split chimeric reporter proteins containing both *Renilla* luciferase (RL) and green fluorescent protein (GFP) variants [17,18]. DSP1-7 has the structure RL_1–155_-Ser-Gly-Gly-Gly-Gly-GFP_1–156_. DSP8-11 has the structure Met-GFP_157–231_ -Gly-Gly-Gly-Gly-Ser- RL_156–311_. RL and GFP become active only when DSP1-7 associates with DSP-8-11 (Supplementary Materials, Figure S1a). The DSP assay scores the degree of membrane fusion between the cells expressing DSP1-7 and the cells expressing DSP8-11 quantitatively according to RL activity in the presence of a membrane-permeant substrate, EnduRen. Since the assay does not involve the use of live viruses, high levels of biological containment essential for experiments involving live viruses are not required [19,20].

Here, we describe the development of a DSP-based cell–cell fusion assay for flaviviruses. Flaviviruses enter host cells via endocytosis; the endosomal environment provides not only the lower pH necessary for conformational changes in the E protein but also optimum membrane composition rich in acidic lipids. These two factors are essential for E protein-mediated membrane fusion [21,22]. We reconstituted the endosomal environment for flavivirus membrane fusion by employing mosquito-derived C6/36 cells, which possess an acidic lipid-rich plasma membrane [23]. Effector cells expressing E protein with DSP1-7 and target cells expressing E protein with DSP8-11 were mixed in a low pH culture medium (Supplementary Materials, Figure S1b). This allowed us to monitor membrane fusion mediated by the E proteins of various Flaviviruses including DENV1, DENV2, ZIKV, JEV, TBEV, YFV, and WNV. Additionally, we optimized our system to allow high-throughput screening (HTS) of potential fusion inhibitors against the E proteins of various flaviviruses. The screening of 1017 FDA-approved drugs using the ZIKV E protein-dependent fusion assay identified atovaquone, an antimalarial agent [24,25], with subsequent assays revealing that atovaquone efficiently suppressed the infection of ZIKV and four distinct serotypes of DENV in both mammalian and mosquito-derived cells in vitro.

## 2. Materials and Methods

### 2.1. Cell Lines and Reagents

C6/36 (ATCC CRL-1660) and Vero (ATCC CCL-81) cells were purchased from the American Type Culture Collection (ATCC; Manassas, VA, USA). Madin–Darby canine kidney (MDCK) cells were kindly provided by Dr. Hideki Hasegawa (National Institute of Infectious Diseases, Tokyo, Japan). Cells were maintained in Eagle Minimum Essential Medium (EMEM; Wako Pure Chemical Co., Osaka, Japan) containing 10% fetal bovine serum (FBS) at 28 °C for C6/36 cells and 37 °C for Vero and MDCK cells. 293FT cells (R70007; Thermo Fisher Scientific, Waltham, MA, USA) were maintained in Dulbecco’s modified Eagle’s medium (DMEM) containing 10% fetal bovine serum (FBS). Plasmid transfection of C6/36 cells was performed using FlyFectin (OZ Biosciences, San Diego, CA, USA) according to the manufacturer protocol. Plasmid transfection of 293FT cells was performed using TurboFect (Thermo Fisher Scientific) according to the manufacturer protocol. EMEM at a specific pH was prepared using EMEM (Sigma-Aldrich, St. Louis, MO, USA) containing MES (Dojindo, Kumamoto, Japan) and 0.1 M sodium hydroxide (Wako, Tokyo, Japan). The FDA-approved drug library (L1300) was purchased from Selleck (Houston, TX, USA) and dissolved in DMSO to a final concentration of 100 μM. The tested drugs were described previously [15]. Atovaquone (Tokyo Chemical Industry, Tokyo, Japan) was dissolved in DMSO to a concentration of 10 mM. NH_4_Cl (Wako) was dissolved in H_2_O to a concentration of 2 M. Neutralizing antibodies against pan-flavivirus (4G2), DENV, and ZIKV (ZKA78 and ZKA64) activities were purchased from Absolute Antibody (Boston, MA, USA).

### 2.2. Construction of Expression Vectors

The DENV1 (KM204119) and DENV2 (KU725663.1) prME-encoding plasmids pCB-DENV1 and pCB-DENV2, respectively, were kindly provided by Dr. Wei-Kung Wang (University of Hawaii, Honolulu, HI, USA), and synthetic DNA corresponding to prME from ZIKV (KX830960.1), JEV (NC_001437.1), TBEV (MK922615.1), YFV (U17066.1), and WNV (GU246644.1) was obtained from Taihe Biotechnology Co. Ltd. (Beijing, China). All cDNAs encoding prME and the split reporter proteins, including DSP1-7 and DSP8-11, were cloned downstream of the A. aegypti polyubiquitin promoter (pUb) in pGL3-Pub (#52891; Addgene, Cambridge, MA, USA). For 293FT cells, DENV1 prME cDNA was cloned downsteam of the CMV promoter in pRK5 (Genentech, South San Fransisco, CA, USA).

### 2.3. DSP Assay in the 384-Well Format

Two days before the DSP assay, C6/36 cells were seeded in either 24-well culture plates (2 × 10^5^ cells/250 µL) or 100-mm-diameter culture dishes (2 × 10^6^ cells/4 mL). One day before the assay, cells were transfected as follows. For the 24-well culture plates, 1 µg pUb-prME and 2 µg pUb-DSP1-7 or pUb-DSP8-11 plasmid were mixed with 2 µL FlyFectin in 50 µL Opti-MEM (Thermo Fisher Scientific, Waltham, MA, USA). For the 100-mm-diameter culture dishes, 16 µg pUb-prME and 32 µg pUb-DSP1-7 or pUb-DSP8-11 were mixed with 32 µL FlyFectin in 800 µL Opti-MEM. After a 20-min incubation at room temperature, cells were transfected with the plasmid mixture to express prME along with DSP1-7 or DSP8-11. At 2 h before the DSP assay, cells were treated with 6 µM EnduRen (Promega, Madison, WI, USA), which allows esterases inside of the cell to convert EnduRen to coelenterazine, which is an actual substrate for RL. Effector cells expressing prME and DSP1-7 and target cells expressing prME and DSP8-11 were detached from the culture plate by pipetting and mixed on ice. After centrifugation (440 *g* for 5 min at 4 °C), the supernatant was replaced with 600 µL or 9.6 mL of EMEM (pH 7.4 or 6.5) for 24-well culture plates or 100-mm-diameter culture dishes, respectively. Each FDA-approved drug (1 µL, *n* = 1 in HTS, *n* = 3 in each drug analysis) dissolved in DMSO was added to the 384-well plates (Greiner Bioscience, Frickenhausen, Germany) using a 12-stage workstation (Biotech, Tokyo, Japan). Then, the mixture of effector and target cells (100 µL; pH 7.4 or 6.5) was added to the wells using a Multidrop dispenser (Thermo Fisher Scientific). After incubation at 28 °C for 1 h, RL activity was measured using a CentroXS3 microplate reader (Berthold Technologies, Oak Ridge, TN, USA).

### 2.4. Western Blot

Western blot analysis was performed as described previously [26], with some variations. Briefly, prME-transfected C6/36 cells and ZIKV-infected Vero cells were lysed using non-reducing sample buffer (25 mM Tris-HCl (pH 6.8), 10% glycerol, and 2% SDS) and boiled at 100 °C for 10 min, because the anti-pan-flavivirus E protein (4G2) antibody is only capable of recognizing its epitope under non-reducing conditions. Horseradish peroxidase-conjugated anti-mouse IgG (GE Healthcare, Madison, WI, USA) was used as a second antibody.

### 2.5. Flow Cytometric Analysis

DENV1 or ZIKV prME-expressing cells were detached from culture plates by pipetting. For permeabilization, cells were treated with 0.1% Triton X-100 in phosphate-buffered saline (PBS) in the absence of Ca^2+^ and Mg^2+^ (PBS(−)) for 15 min at room temperature and washed with fluorescence-activated cell sorting (FACS) buffer (0.1% FBS in PBS(−)). Cells were incubated with 1 µg/mL 4G2 in FACS buffer at 4 °C overnight, followed by washing with FACS buffer and staining with 10 μg/mL Alexa-Fluor488 goat anti-mouse IgG (Life-Tech, Inc., Houston, TX, USA) at 4 °C for 30 min. Then, cells were washed with FACS buffer and analyzed using a FACSVerse flow cytometer (BD Biosciences, San Jose, CA, USA).

### 2.6. Viral Infection Assay

ZIKV strain VR-84 was purchased from ATCC. DENV1 (02-17-HuNIID), DENV2 (TL30-05), DENV3 (CH-53489), and DENV4 (10-19/HuNIID) were kindly provided by Dr. Chang-Kweng Lim (National Institute of Infectious Diseases, Tokyo, Japan). Influenza virus strain A/Puerto Rico/8/34 was kindly provided by Dr. Hideki Hasegawa. The effect of atovaquone on the infection of viruses was measured as follows. The target Vero and C6/36 cells for ZIKV and DENV1-4 or MDCK cells for influenza virus were pretreated with atovaquone for 1 h, followed by the addition of viruses to each target cell culture at an MOI of 0.01. The cells were incubated for 1 h and then washed three times with PBS(−). The infected cells were further cultured in 2% FBS DMEM for ZIKV and DENV1-4 or Opti-MEM containing acetyltrypsin (10 mg/mL) for influenza virus and in the presence of various concentrations of atovaquone. At 48 h after ZIKV and DENV1-4 infection and 24 h after influenza virus infection, total RNA was extracted using TRIZOL reagent (Life-Tech, Inc.), and viral RNAs were measured by real-time PCR using the appropriate primers. To analyze the role of atovaquone in viral entry and post-entry steps, the following experiments were performed. For the pretreatment experiment (Pr), Vero cells were pretreated with 3 µM atovaquone or 20 mM NH_4_Cl for 1 h before challenge with ZIKV. Then, the cells were incubated with ZIKV (MOI = 0.1) for 1 h in the presence of inhibitors and then washed three times with PBS(−). The infected cells were further cultured for 24 h in 2% FBS DMEM without inhibitors. For the post-treatment experiment (Po), Vero cells were incubated with ZIKV (MOI = 0.1) for 1 h in the absence of inhibitors and then washed three times with PBS(−). The infected cells were further cultured for 24 h in 2% FBS DMEM with inhibitors. For the Pr+Po experiment, pretreatment incubation, infection, and post-treatment incubation were performed in the presence of inhibitors. At 24 h after ZIKV infection, total RNA was extracted using TRIZOL reagent (Life-Tech, Inc.), and viral RNAs were measured by real-time PCR using the appropriate primers. The primers used were ZIKV NS5, 5′-GGC CAC GAG TCT GTA CCA AA-3′ and 5′-AGC TTC ACT GCA GTC TTC C-3′; and DENV1-4 C-prM, 5′-TCA ATA TGC TGA AAC GCG CGA GAA ACC G-3′ and 5′- TTG CAC CAA CAG TCA ATG TCT TCA GGT TC-3′; and influenza virus NP, 5′-AGA ACA TCT GAC ATG AGG AC-3′ and 5′-GTC AAA GGA AGG CAC GAT C-3′.

### 2.7. Trypan Blue Dye-Exclusion Assay

At 72 h after atovaquone treatment, cells in the culture supernatant and those detached from the substrate by trypsinization were mixed, and the resulting mixed cell suspension was centrifuged for 5 min at 400× *g*. The cell pellet was then resuspended in PBS(−) and mixed with an equal volume of 0.3% Trypan blue. Unstained (living) and stained (dead) cells were counted using a hemocytometer (03-202-1 Erma, Tokyo, Japan).

### 2.8. Statistical Analysis

Statistically significant differences between the mean values were determined using a two-tailed Student’s *t* test with Bonferroni correction. All data represent three independent experiments, and values represent the mean ± standard deviation (s.d.), with a *p* < 0.05 considered statistically significant.

## 3. Results

### 3.1. Establishment of a Membrane-Fusion Assay for Flavivirus E Proteins Based on the DSP Reporter in Mosquito-Derived C6/36 Cells

To develop a DSP-based cell-fusion assay for flavivirus E proteins using the DENV serotype 1 (DENV1) E protein as an initial model [27], we first tested whether a 293FT cell-based assay system could be constructed similar to our previous HIV-1, MERS-CoV, and SARS-CoV-2 system [15,16,28]. 293FT cells are suitable for cell–cell fusion assays based on the co-cultivation of effector and target cells because of their high degree of transfectability and smooth detachment from culture plates. However, in agreement with reports citing the requirement for acidic lipids in E protein-mediated membrane fusion [22], we failed to observe increased luciferase activity indicative of cell fusion. In addition to the difference in lipid content, our data confirmed results from previous reports [22,29] showing that the transport of E proteins to the 293FT cell surface is inefficient, despite their relatively high level of intracellular expression (Supplementary Materials, Figure S2). Although acidic lipids can be complemented exogenously [22], this might introduce artifacts derived from chemical interactions between the exogenous lipids and test compounds. Since mosquito cells serve as hosts for the natural replication of many flaviviruses, and the mosquito-derived cell line C6/36, which contains acidic phospholipids in its plasma membrane [23], has been used to propagate DENV [30], we next used C6/36 cells to establish the E protein-mediated cell-fusion assay.

**Figure 1:**
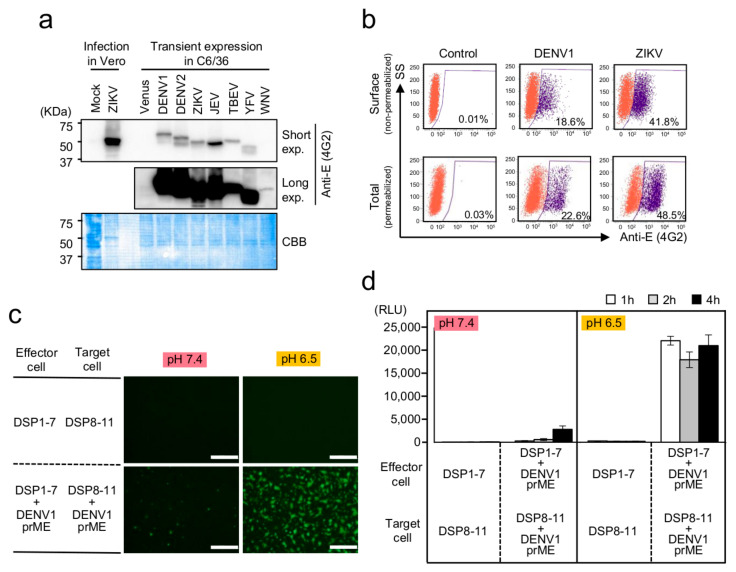
Establishment of a membrane-fusion assay system for flavivirus E proteins based on the dual split protein (DSP) reporter in mosquito-derived C6/36 cells. (**a**) Expression of the E proteins of various flaviviruses. Western blot analysis of Zika virus (ZIKV)- or mock-infected Vero cells, and C6/36 cells transfected with the expression vector for the Venus or prME proteins of various flaviviruses under the control of the *A. aegypti* ubiquitin promoter. E proteins were detected using a mouse anti-E-protein antibody (4G2) that binds to a conserved epitope found on E proteins of the Flavivirus genus. Short and long exposures of the same blot (top and middle). Coomassie Brilliant Blue staining (bottom) of the membrane showing the amount of loaded protein. (**b**) Total expression of E protein (Total) and that on the cell surface (Surface) was determined by fluorescence-activated cell sorting (FACS) analysis using non-permeabilized and permeabilized cells. (**c**,**d**) Cell–cell membrane fusion was analyzed using DSP reconstitution by detecting (**c**) green fluorescent protein (GFP) and (d) Renilla luciferase (RL) activities derived from the association of DSP1-7 and DSP8-11. GFP signals were detected at 2 h after mixing of the effecter and target cells. RL activities were measured at the indicated time points after mixing of the effecter and target cells. Membrane fusion was induced by lowering the pH of the medium from pH 7.4 to pH 6.5. Scale bars, 500 µm. RLU: relative light unit.

To achieve the sufficient expression of E and DSP proteins in C6/36 cells, we sought an appropriate promoter for use in C6/36 cells. Since the ubiquitin gene is constitutively expressed in mosquito cells, we constructed expression vectors harboring prME genes derived from various flaviviruses and the reporter proteins DSP1-7 and DSP8-11 under the control of the ubiquitin promoter from *Aedes aegypti* [31]. Western blot using the anti-pan-flavivirus E protein (4G2) antibody revealed that E proteins from several flaviviruses were easily detected in C6/36 cells (Figure 1a). Furthermore, significant portions of the E proteins of DENV1 and ZIKV were expressed on the surface of C6/36 cells (Figure 1b). Then, we checked whether the E protein expressed on the cell surface can induce cell–cell fusion. The effector cells transfected with the expression vectors for the E protein of each flavivirus and DSP1-7 and the target cells transfected with the expression vector for the same E protein and DSP8-11 were co-cultured at various pH values between 7.4 and 5.0. Luciferase activities were significantly increased at lower pH, although their pH dependencies and absolute values varied among viruses, which may reflect virus-specific differences in infectivity and pathogenesis (Supplementary Materials, Figure S3). We chose E protein of DENV1 and ZIKV and fixed the pH to 6.5 to validate our DSP assay system, since we intended to search for inhibitors for DENV and ZIKV infection, and all the viruses tested here showed maximal or submaximal activities of luciferase at pH 6.5. Co-culture of the effector cells expressing DENV1 E and DSP1-7 with the target cells expressing DENV1 E and DSP8-11 resulted in significant levels of GFP signal and RL activity, which reached a plateau at 1 h of co-culture, in a low-pH- and E-protein-dependent manner (Figure 1c and d). Additionally, the microscopy of cells co-cultured for 1 h revealed that a significant number of fused cells were observed only in the presence of E protein at low pH (Supplementary Materials, Figure S4). These results indicated that the combination of C6/36 cells and the ubiquitin-promoter-driven expression system enabled the DSP assay to quantify cell–cell fusion induced by the E protein of various Flaviviruses.

**Figure 2:**
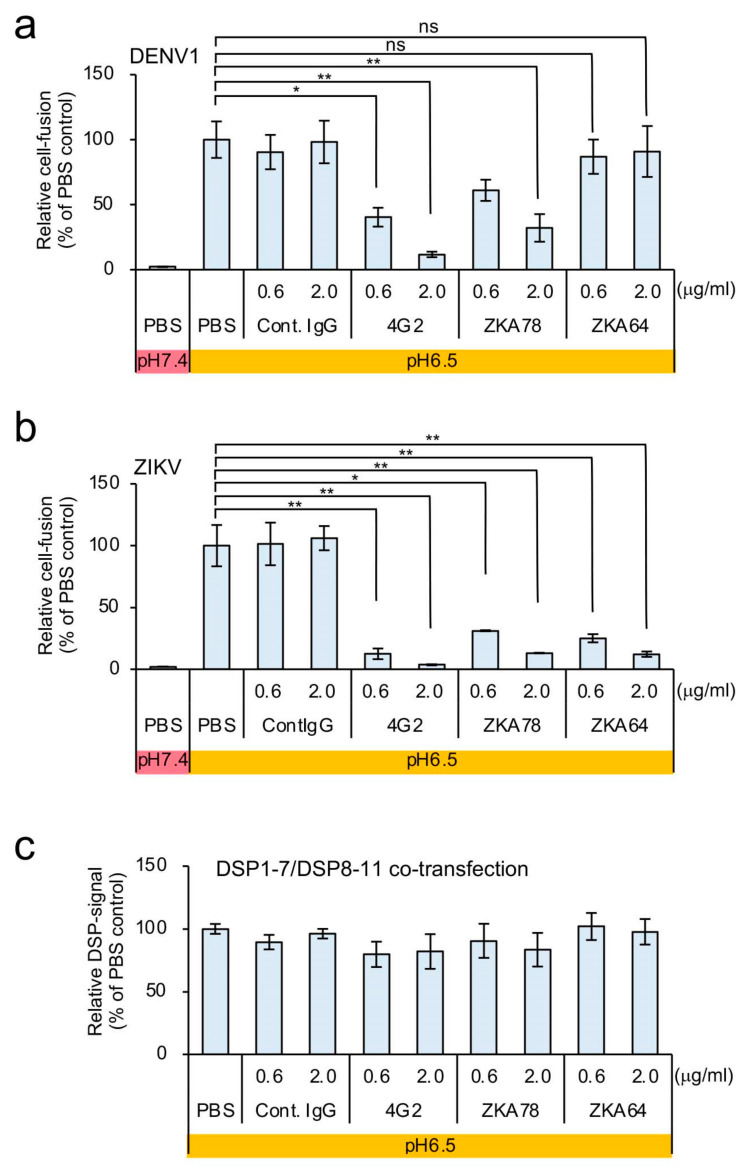
Effect of anti-E-protein monoclonal antibodies on the DSP assay in C6/36 cells expressing the dengue virus 1 (DENV1) and ZIKV E proteins. (**a**,**b**) Neutralizing antibodies were added during co-culture of the effector and target cells in the DSP assay, including the mouse monoclonal anti-4G2 antibody recognizing various flavivirus E proteins and mouse monoclonal antibodies ZKA78 (cross-reactive with the DENV E protein) and ZKA64 recognizing the ZIKV E protein. Relative cell-fusion value was calculated by normalizing the RL activity for each antibody to the RL activity of the control assay (phosphate-buffered saline (PBS); set to 100). (**a**) Cells expressing the DENV1 E protein and (**b**) those expressing the ZIKV E protein. (**c**) Each antibody was added to C6/36 cells co-expressing DSP1-7 and DSP8-11 in order to evaluate its direct non-specific inhibitory effects on RL activity. Relative DSP-signal value was calculated by normalizing the RL activity for each antibody to the RL activity of the control assay (PBS; set to 100). Statistical analysis was performed using Student’s t test with Bonferroni correction. * *p* < 0.05; ** *p* < 0.01. ns: no significant difference.

To confirm the dependence of the DSP signal on cell fusion by the E protein, we used various anti-E protein monoclonal antibodies. The mouse monoclonal antibody 4G2 recognizes various flavivirus E proteins (Figure 1a). Additionally, mouse monoclonal antibodies ZKA78 and ZKA64 were isolated from ZIKV-infected individuals, and ZKA78 but not ZKA64 is cross-reactive with the DENV E protein [32]. All of these antibodies exhibit neutralizing activity against E protein [33]. Compared with a control antibody, RL signals were significantly reduced following the introduction of 4G2 and ZKA78 but not ZKA64 in the DSP-monitored fusion mediated by DENV1 E protein (Figure 2a). Moreover, we found that the DSP signals derived from ZIKV E were specifically blocked by all of these antibodies (Figure 2b), and that none of the antibodies affected the RL activities derived from cells co-transfected with DSP1-7 and DSP8-11 (Figure 2c). These specific neutralizing-antibody-dependent reductions in RL activity (Figure 2a,b) showed that our assay faithfully reconstitutes E-protein-mediated cell fusion.

### 3.2. Optimization of the C6/36-Based DSP Assay for HTS of Membrane-Fusion Inhibitors

Then, we tried to adapt the DSP assay to a 384-well format in order to enable HTS of potential inhibitors of cell-fusion mediated by the E protein of various flaviviruses. By adjusting several parameters, including the numbers of effector and target cells and the duration of co-cultivation, we established suitable conditions for HTS and calculated the Z factor to verify the reproducibility and reliability of our assay in the 384-well format [34]. The obtained Z factors for the assay were >0.5, thereby confirming the reproducibility of the assay (Table 1 and Supplementary Materials, Figure S5).

### 3.3. Identification of Atovaquone as a Potent Inhibitor of ZIKV E-Protein-Induced Membrane Fusion

**Figure 3:**
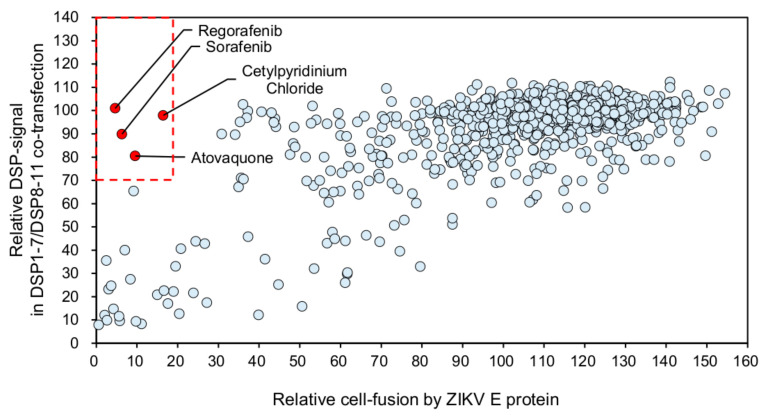
HTS of 1017 FDA-approved drugs in the DSP assay using the ZIKV E protein. The X-axis shows the relative cell-fusion value in the presence of each drug (1 µM in DMSO), *n* = 1. The relative cell-fusion value was calculated by normalizing the RL activity for each drug to the RL activity of the control assay (DMSO alone; set to 100). The Y-axis shows the relative DSP signal, which indicates the non-specific direct inhibitory effect of each drug on RL activity. The relative DSP signal value was calculated by normalizing the RL activity of the cells co-expressing DSP1-7 and DSP8-11 with each drug to the RL activity of the cells co-expressing DSP1-7 and DSP8-11 with DMSO alone (set to 100). Each dot represents an individual drug. The data shown in this figure were obtained from a single experiment. Red dots in the red-dashed box indicate positive hits (>80% inhibition of the relative cell-fusion value in the X-axis and <30% inhibition of the relative DSP-signal value in the Y-axis. Drug names of positive hits are indicated.

Using our established screening system for ZIKV E-protein-mediated cell fusion, we tested 1017 FDA-approved drugs in order to identify membrane-fusion inhibitors (Figure 3). We judged a drug as a positive hit if the fusion-dependent RL activities fell below 20% (Figure 3, X-axis), while the RL activities derived from DSP1-7/DSP8-11 co-transfection remained above 70% (Figure 3, Y-axis). We selected four drugs that met our criteria: regorafenib, sorafenib, cetylpyridinium chloride, and atovaquone (Figure 3, red dots in the red-dashed box indicating the area of Y > 70 and X < 20).

**Figure 4:**
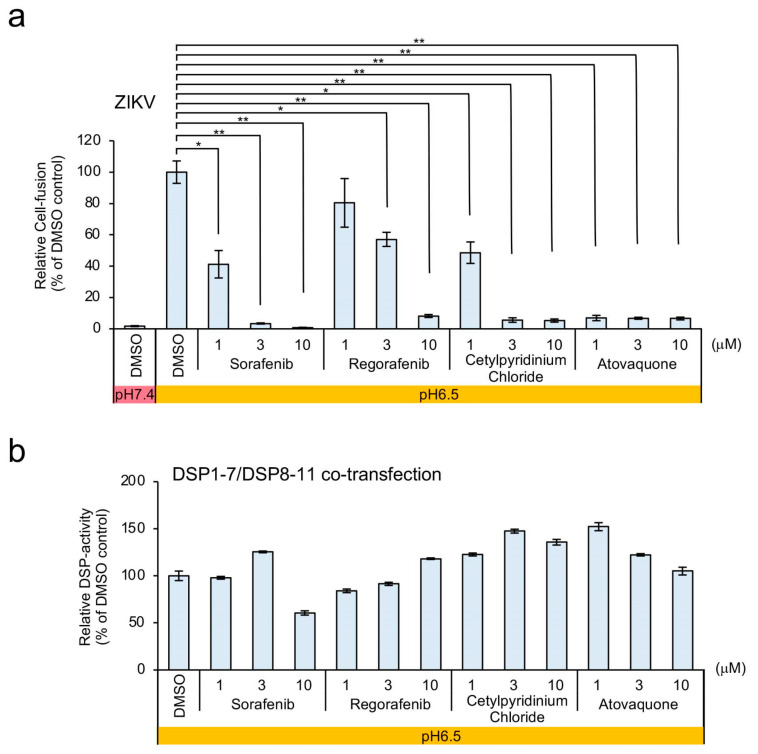
Evaluation of the dose response associated with the four positive hits on HTS based in ZIKV E-protein-mediated membrane fusion using the DSP assay. (**a**) DSP assay evaluation of each candidate drug was performed following the induction of membrane fusion at pH 6.5 and in the presence of different concentrations of the drugs. The relative cell-fusion value was calculated by normalizing the RL activity for each drug to the RL activity of the control assay (DMSO alone; set to 100). (**b**) The non-specific direct effect of each drug on RL activity was evaluated following the addition of each drug to cells co-expressing DSP1-7 and DSP8-11. The relative DSP-signal value was calculated by normalizing each RL activity to the RL activity of the control assay (DMSO alone; set to 100). Statistical analysis was performed using Student’s t test with Bonferroni correction. *p < 0.05; **p < 0.01.

Dose–response analyses revealed that atovaquone showed the strongest inhibition among the four candidates (Figure 4a) in the absence of any non-specific inhibitory effects on RL activity (Figure 4b). Therefore, we chose atovaquone for further analysis.

**Figure 5:**
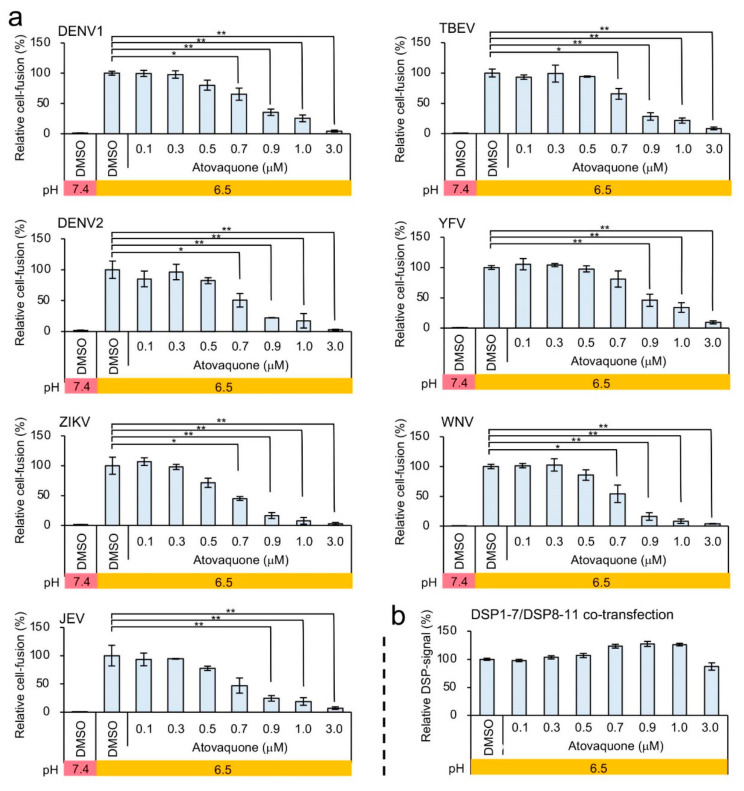
The effect of atovaquone on DENV1, dengue virus (DENV2), ZIKV, Japanese encephalitis virus (JEV), tick-borne encephalitis virus (TBEV), yellow fever virus (YFV) and West Nile virus (WNV) E-protein-mediated cell fusion. (**a**) Atovaquone was tested at different concentrations, with low-pH conditions (pH 6.5) used to induce membrane fusion. The relative cell-fusion value was calculated by normalizing each RL activity to the RL activity of the control assay (DMSO alone; set to 100). (**b**) The non-specific inhibitory effect of atovaquone on RL activity was evaluated as described in Figure 4b. The relative DSP-signal value was calculated by normalizing each RL activity to the RL activity of the control assay (DMSO alone; set to 100). Statistical analysis was performed using Student’s t test with Bonferroni correction. * *p* < 0.05; ** *p* < 0.01.

To investigate the potential mechanism of inhibition of ZIKV E-mediated membrane fusion by atovaquone, we evaluated the effect of atovaquone on other E proteins of flaviviruses. Similar to the inhibition profile of ZIKV E-mediated fusion, membrane fusion by the E proteins of various flaviviruses was inhibited by atovaquone (Figure 5a,b). Moreover, the number of fused cells mediated by DENV1 E at low pH was also significantly reduced by atovaquone treatment without affecting the medium pH and surface E protein expression (Supplementary Materials, Figures S6 and S7). These results strongly suggested that atovaquone targets molecules or processes common to membrane fusion induced by the E proteins of various flaviviruses.

### 3.4. Efficient Inhibition of ZIKV and DENV1-4 Infection in Mosquito and Mammalian Cells by Atovaquone

**Figure 6:**
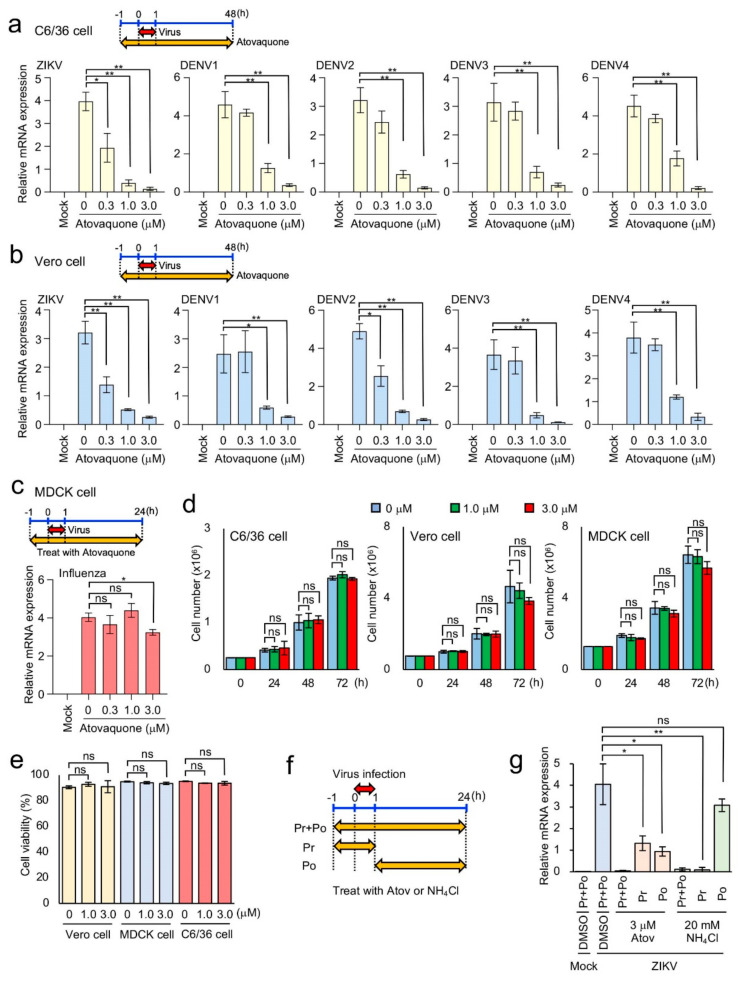
The effect of atovaquone on ZIKV, DENV1-4, and influenza virus infection in vitro. (**a**–**c**) Effects of atovaquone on viral RNA amplification of ZIKV and DENV1-4 in C6/36 cells (**a**) and Vero cells (**b**) and influenza virus in MDCK cells (**c**). Cells for ZIKV, DENV, and Madin–Darby canine kidney (MDCK) cells for influenza virus were pretreated with various concentrations of atovaquone for 1 h before challenge with each virus (MOI = 0.01). The cells were incubated with each virus for 1 h and then washed three times with PBS(−). The infected cells were further cultured in 2% fetal bovine serum (FBS) DMEM for ZIKV and DENV or Opti-MEM containing acetyltrypsin (10 mg/mL) for influenza virus and in the presence of various concentrations of atovaquone. At 48 h after ZIKV and DENV infection and 24 h after influenza virus infection, total RNA was extracted. The relative amount of viral RNA in the cells was quantified by real-time PCR. The result shown is representative of duplicate experiments. (**d**) Effects of atovaquone on the proliferation of C6/36, Vero, and MDCK cells. Cells were treated with various concentrations of atovaquone under the same conditions as described in (**a**–**c**) without viruses. At the indicated time after atovaquone treatment, the cell number was counted. (**e**) The effect of atovaquone on the viability of C6/36, Vero, and MDCK cells. Treatment of cells with atovaquone was performed as described in (**d**). At 72 h after atovaquone treatment, cell viability was measured by trypan blue dye-exclusion assay. (**f**) Schematic shows the infection assay in (**g**). For the pretreatment (pr), Vero cells were pretreated with 3 μM atovaquone or 20 mM NH_4_Cl for 1 h before challenge with ZIKV. The cells were incubated with ZIKV (MOI = 0.1) for 1 h in the presence of inhibitors and then washed three times with PBS(−). The infected cells were further cultured for 24 h in 2% FBS DMEM without inhibitors. For the post-treatment (po), Vero cells were incubated with ZIKV (MOI = 0.1) for 1 h in the absence of inhibitors and then washed three times with PBS(−). The infected cells were further cultured for 24 h in 2% FBS DMEM with inhibitors. For the Pr+Po experiment, pretreatment incubation, infection, and post-treatment incubation were performed in the presence of inhibitors. Atov: Atovaquone. (**g**) The effect of atovaquone and NH_4_Cl on viral entry and post-entry step. Statistical analysis was performed by Student’s t test with Bonferroni correction. * *p* < 0.05; ** *p* < 0.01. ns: no significant difference.

Our DSP assay showed the inhibitory effect of atovaquone on E-mediated membrane fusion by ZIKV and DENV. Then, we examined the effect of atovaquone on the infection of actual flaviviruses including ZIKV and DENV1-4 in C6/36 and Vero cells. Atovaquone was added 1 h prior to 1 h infection with ZIKV or DENV1-4. Forty-eight hours after infection, total RNA was extracted to measure the amounts of viral mRNA. Similar experiments using MDCK cells were performed using influenza virus as a control in order to measure viral NP mRNA in cells infected with virus. The addition of atovaquone to the culture medium resulted in a significant reduction in ZIKV and DENV1-4 mRNA expression in both C6/36 cells (Figure 6a) and Vero cells (Figure 6b; IC_90_ = 2.1 μM for ZIKV, 2.5 μM for DENV1, 1.7 μM for DENV2, 1.6 μM for DENV3 and 2.5 μM for DENV4). By contrast, atovaquone addition to the influenza virus infection did not significantly alter the levels of viral NP mRNA (Figure 6c). Furthermore, we observed minimal effects on the growth and viability of C6/36, Vero, and MDCK cells at atovaquone concentrations of up to 3 μM (Figure 6d,e), which is higher than the IC_90_ observed in viral-infection experiments (Figure 6b). These results indicated that atovaquone potently inhibited ZIKV and DENV infection but not influenza virus in mammalian cells in vitro. Although influenza possesses a class I fusion protein, it enters target cells by endocytosis and subsequent membrane fusion triggered by low pH in endosomes. Our data on its insensitivity to atovaquone may suggest that its fusion mechanism differs from that of ZIKV and DENV1-4 E proteins, which belongs to the class II fusion proteins.

Atovaquone was reported to block ZIKV infection by blocking pyrimidine synthesis [35], which is distinct from our finding that atovaquone inhibits the viral entry step. Therefore, we investigated the effect of atovaquone treatment before and during infection (Pr; pretreatment) and that after infection (Po; post-treatment) on viral replication (Figure 6f). The treatment with endosomal acidification inhibitor, NH_4_Cl, in the Pr experiment completely blocked viral replication, while that in the Po experiment did not (Figure 6g). Under otherwise identical conditions, the treatment with atovaquone in the both the Pr and Po experiments partially but significantly inhibited viral replication (Figure 6g). Envelope fusion-mediated viral entry is likely to occur during 1 hr of viral infection, which is supported by our data that NH_4_Cl treatment in the Pr experiment completely blocked viral replication. Therefore, atovaquone likely blocks both envelope fusion and pyrimidine synthesis.

## 4. Discussion

In this study, we described the establishment of a quantitative assay for cell-fusion mediated by the E protein of members of the Flavivirus genus, including DENV1, DENV2, ZIKV, JEV, TBEV, YFV, and WNV. We adapted the DSP assay system, which has been successfully utilized to monitor cell fusions mediated by fusion proteins of HIV-1, MERS-CoV, and SARS-CoV-2 [15,16,19], for use in C6/36 cells derived from *A. albopictus*. Our assay system mimics the membrane fusion between the flavivirus envelope and the endosomal membrane by providing an acidic lipid-rich plasma membrane present in C6/36 cells [22] and a low-pH environment controlled by manipulating the culture media, thereby allowing an evaluation of membrane-fusion activity independent of endocytosis. By altering the pH of the incubation media, we preliminarily showed that the optimal pH necessary for flavivirus E-protein-mediated membrane fusion varied among members of the Flavivirus genus. Further analysis of the pH-dependency differences using our fusion system might enable the elucidation of differences in the timing of viral entry during endocytosis among various flaviviruses.

Our DSP assay offers a number of advantages for its use in performing HTS. It is robust, easy to use under normal laboratory conditions, and rapid (≈2 h), and allows quantitative assessment giving it an advantage over other methods relying upon cytopathic effects, such as syncytia formation [36]. Furthermore, the DSP assay does not require burdensome image analysis, which is necessary in dye-transfer assays [37] or counting cells double-labeled with green and red fluorescence proteins [22]. Since our DSP assay quantifies cell–cell fusion as luciferase activity, whose amount increases almost linearly with every single fusion event, the DSP assay is likely to be more accurate than other existing fusion assay systems. Since the DSP assay depends solely on pre-existing reporter proteins, compounds that inhibits DNA, RNA, or protein synthesis scarcely generate false-positives. Furthermore, the false-positive compounds that affect RL activity can easily be ruled out by applying the candidate compounds directly to the DSP1-7/DSP8-11 co-expressing cells. Among the viral proteins, only prME is necessary for the assay, thereby eliminating the necessity to potentially generate live infectious viruses, which require high levels of biological containment.

Here, we screened a library of the FDA-approved drugs. This approach involving retargeting approved medicines (i.e., “drug repurposing”) can significantly shorten the time required for the development of effective therapeutics, because clinical data regarding their safety have been accumulated [38]. Although our screening was performed once and its reproducibility was not confirmed, hit compounds were validated by the subsequent dose–response experiments. Our results identified atovaquone, previously used for the treatment and prophylaxis of malaria caused by Plasmodium and pneumocystis pneumonia caused by the fungus Pneumocystis jirovecii [39,40]. A previous human study reported that the standard manufacturer-recommended dose for the oral administration of atovaquone results in blood atovaquone concentrations ranging from 10 to 30 µM for several days [41]. Our results showed that IC_90_ values for in vitro infection of ZIKV and DENV1-4 are 1.6–2.5 µM, suggesting that the currently recommended dosing of atovaquone would likely be effective at preventing ZIKV and DENV infection in vivo. However, this suggestion should be tested by animal infection experiments prior to clinical studies. Since the geographical distributions of vector insects associated with ZIKV, DENV, and malaria overlap [42], atovaquone administration might be enormously beneficial for people living in endemic areas, which could be validated by appropriate retrospective cohort studies.

Atovaquone inhibits binding between ubiquinone and the cytochrome bc1 complex in the inner mitochondrial membrane, thereby blocking ATP synthesis [43,44]. Atovaquone preferentially inhibits the respiratory chain in P. jirovecii and Plasmodium, although it does this >100-fold less efficiently in humans [40,45]. Despite clear identification of its target in the treatment of malaria and pneumocystis pneumonia, we cannot explain the potential inhibitory mechanism of atovaquone on ZIKV E-protein-mediated membrane fusion. Since atovaquone showed a specific inhibition of ZIKV and DENV—but not on influenza virus—replication, it is unlikely that its inhibitory mechanism is mediated by the inhibition of ATP-dependent endocytosis, which is utilized for the entry of these viruses. Given that atovaquone has antiviral potential in both Vero and C6/36 cells and that it blocked membrane fusion induced by the E proteins of various flaviviruses, its target could be either a molecule involved in some fundamental function common to mammalian and insect cells or a specific structure in viral prME proteins unique to the flavivirus genus. Further study may reveal a new therapeutic target common to other flaviviruses.

Atovaquone has also been shown to inhibit dihydroorotate dehydrogenase (DHODH), which is an enzyme required for de novo pyrimidine synthesis, leading to the reduction of intracellular nucleotides [43,46,47,48]. Atovaquone was recently reported to block ZIKV infection by blocking pyrimidine synthesis [35]. The authors reported that atovaquone more efficiently blocked ZIKV infection when added at an early time point in infection (up to 4 h post infection) in HEK293T cells, which seems consistent with our finding that atovaquone inhibits the step of membrane fusion, an essential early step of replication. However, they also observed that treatment for only the first 1 h after infection was insufficient. In contrast, we demonstrate here that atovaquone treatment prior to and during infection (Pre experiment) as well as that post infection (Po experiment) significantly inhibits viral replication. Since the endosome acidification inhibitor NH_4_Cl in the Pre experiment almost completely inhibits viral replication, atovaquone could suppress the membrane fusion step in Vero cells in addition to RNA replication. Therefore, the inhibition of both the membrane fusion and RNA replication steps may contribute to the effective suppression of ZIKV and DENV infection by atovaquone. Further investigation is required to elucidate the precise inhibitory mechanism of atovaquone, which should lead to the discovery of new drugs that are more effective than atovaquone.

Based on dose–response results, we focused on atovaquone in this study. However, our screening results identified two tyrosine kinase (TK) inhibitors (regorafenib and sorafenib) and a cationic quaternary ammonium antiseptic reagent (cetylpyridinium chloride) as potential inhibitors of membrane fusion. A previous study reported the involvement of receptor TKs (RTKs) belonging to the Tyro-3, Axl, and Mer family of RTKs in flavivirus infection [49]. ZIKV binds indirectly to Axl via GAS6, a natural ligand of Axl that recognizes phosphatidylserine exposed at the surface of the ZIKV envelope and acts as a bridge between the viral particle and Axl on the host-cell membrane. It is possible that the identified TK inhibitors might inhibit Axl or related proteins in order to block ZIKV E-protein-mediated fusion. Cetylpyridinium chloride is a cationic surfactant that is capable of potentially interacting with acidic lipids at the surface of the host membrane and/or viral envelope to interfere with their critical role in membrane fusion. The use of mosquito-derived C6/36 cells harboring high concentrations of acidic lipids in their plasma membrane (which mimics the endosomal membrane of mammalian cells) for our HTS might have contributed to the selection of these drugs.

In addition to its use for drug screening and evaluating neutralizing antibodies, our results confirmed the efficacy of the DSP assay for use in analyzing mechanisms associated with flavivirus E-protein-mediated membrane fusion. Since our assay works independent of endocytosis, and the assay pH can be specifically controlled exogenously, the membrane-fusion abilities of various E-protein mutants under different pH conditions can be easily evaluated. We believe our assay will be useful for both basic and applied studies of flavivirus E-protein-mediated membrane fusion.

## Figures and Tables

**Table 1:** Reproducibility and reliability of the DSP assay in the 384-well format.

Virus	Signal/Background Ratio	Z Factor
DENV1	136.2	0.76
DENV2	15.4	0.58
ZIKV	35.2	0.82
JEV	390.5	0.75
TBEV	31.1	0.67
YFV	220.4	0.69
WNV	13.0	0.59

Signal/Background ratios and Z factors were calculated based on the results shown in Supplementary Materials, Figure S5.

## Supplementary Materials

The following are available online at https://www.mdpi.com/1999-4915/12/12/1475/s1, Figure S1: Cell-based membrane-fusion assay for flavivirus E proteins using the DSP reporter., Figure S2: Insufficient expression of the DENV1 E protein on the cell surface of 293FT cells., Figure S3: The pH-dependencies of DSP assay using E proteins of various flavivirus., Figure S4: Cell fusion detected in prME-expressing C6/36 cells under low-pH conditions., Figure S5: Validation of DSP-assay efficacy for HTS using flavivirus E proteins., Figure S6: Inhibition of DENV1 E protein-mediated cell fusion by atovaquone., Figure S7: No alteration in medium pH and surface E protein expression levels by atovaquone treatment.
