# Supplementary material for: The Antimalarial Compound Atovaquone Inhibits Zika and Dengue Virus Infection by Blocking E Protein-Mediated Membrane Fusion
Source: Viruses. 2020 Dec 21;12(12):1475. doi: 10.3390/v12121475 (PMC7767512; doi:10.3390/v12121475)
Supplement: Supplementary file 1 [file viruses-12-01475-s001.pdf]

Supplementary Materials

The Antimalarial Compound Atovaquone Inhibits Zika and Dengue Virus Infection by Blocking E Protein-Mediated Membrane Fusion

Mizuki Yamamoto, Takeshi Ichinohe, Aya Watanabe, Ayako Kobayashi, Rui Zhang, Jiping Song, Yasushi Kawaguchi, Zene Matsuda, and Jun-ichiro Inoue

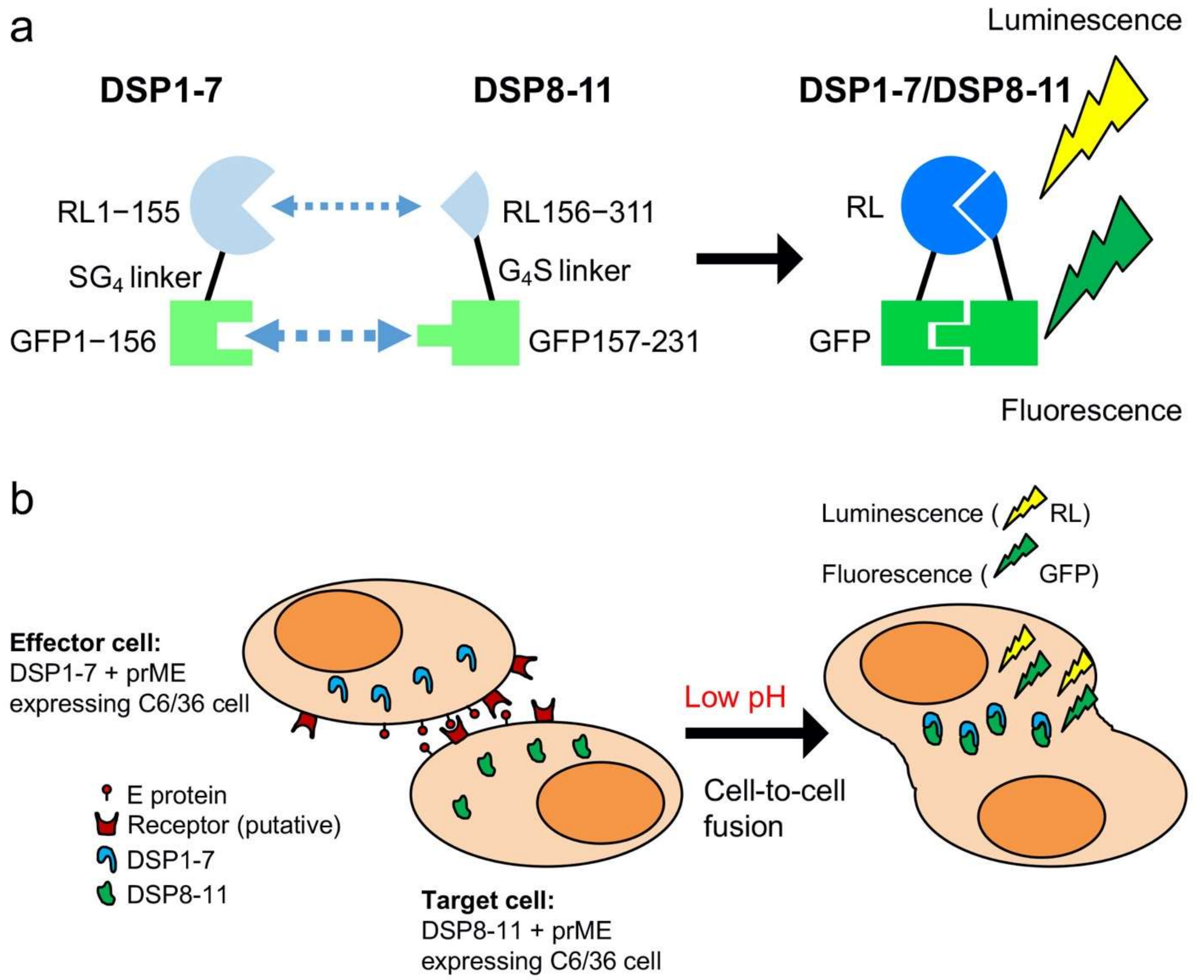

Figure S1 Cell-based membrane-fusion assay for flavivirus E proteins using the DSP reporter. **a** Schematic diagram of split chimeric reporter proteins. DSP1-7 has the structure $RL_{1–155}$-Ser-Gly-Gly-Gly-Gly-$GFP_{1–156}$. DSP8-11 has the structure Met- $GFP_{157–231}$ -Gly-Gly-Gly-Gly-Ser- $RL_{156–311}$. Because $GFP_{1–156}$ contains the first seven sheets, and $GFP_{157–231}$ contains the remaining four sheets, the split proteins were called DSP1-7 and DSP8-11, respectively. DSP1-7 and DSP8-11 reassociate efficiently, resulting in reconstitution of functional RL and GFP to generate luminescent and fluorescent signals, respectively. **b** A method to monitor cell-cell membrane-fusion mediated by the E protein of flaviviruses. Effector cells (C6/36 cells expressing DSP1-7 and prME) and target cells (C6/36 cells expressing DSP8-11 and prME) were co-cultured at pH 6.5 at 28°C. Both GFP (fluorescence) and RL (luminescence) signals were generated following DSP1-7 and DSP8-11 reassociation upon mixture of cells during the assay.

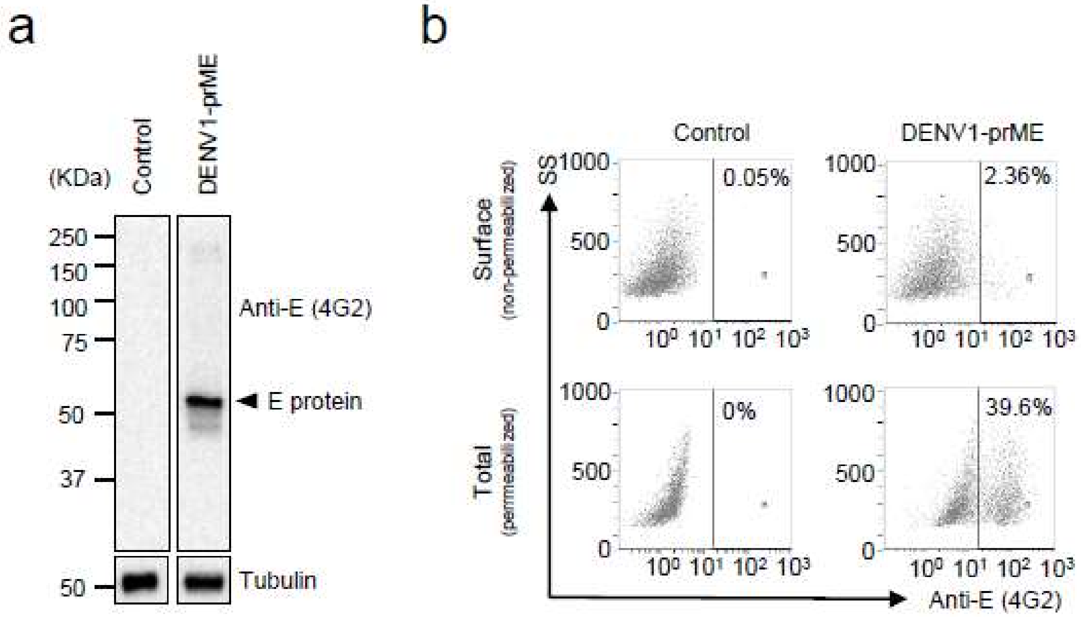

Figure S2 Insufficient expression of the DENV1 E protein on the cell surface of 293FT cells. **a** DENV1 E protein was transiently expressed in 293FT cells, followed by western blot analysis using the anti-E-protein antibody (4G2). An arrowhead denotes the DENV1 E protein. **b** Surface expression of the E protein was analyzed by FACS. Despite strong signals in permeabilized cells, E protein expression on the cell surface was barely detectable in non-permeabilized cells.

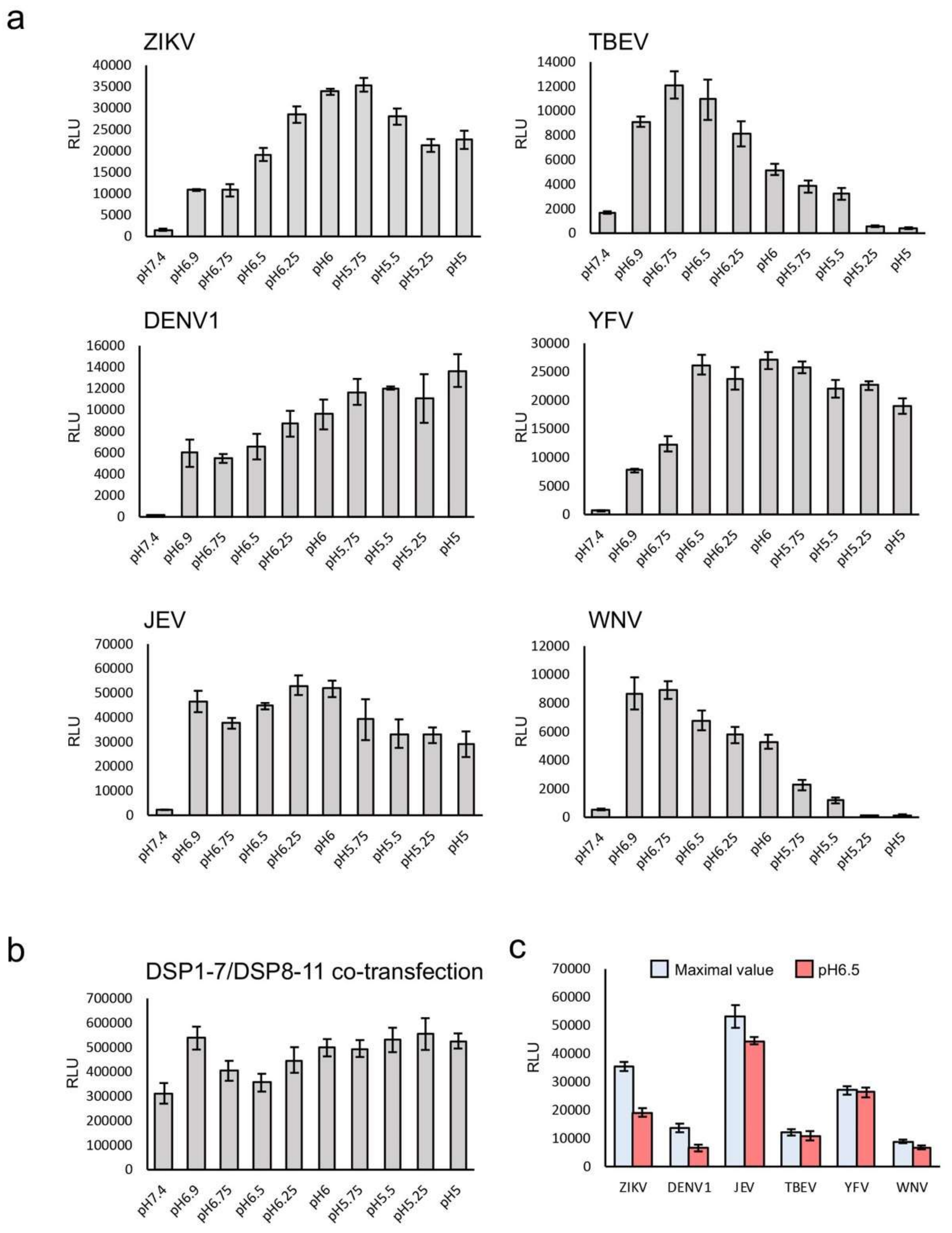

Figure S3 The pH-dependencies of the DSP assay using E proteins of various flavivirus. **a** Effector C6/36 cells expressing E protein of each flavivirus and DSP1-7 and target cells expressing the same E protein as in effector cells and DSP8-11 were mixed. The pH of the medium was changed from 7.4 to the indicated pH conditions. Cells were further incubated for 1 h at 28°C. The relative light unit (RLU) values were measured. **b** C6/36 cells expressing both DSP1-7 and DSP8-11 were cultured for 1hr at the indicated pH, and RLU values were measured. **c** Summary of maximum DSP assay values juxtaposed with their respective values at pH 6.5 (taken from **a)**.

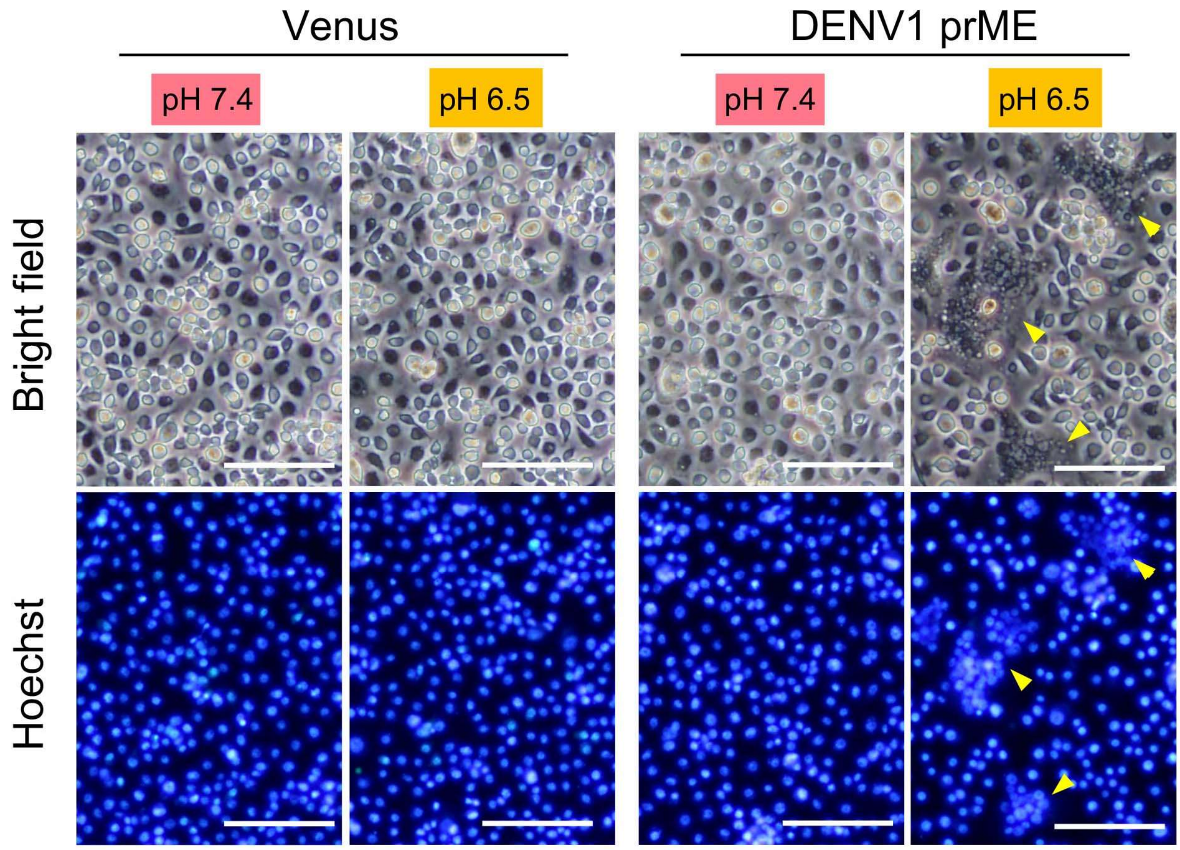

Figure S4 Cell fusion detected in prME-expressing C6/36 cells under low-pH conditions. DENV1 prME and Venus were transiently expressed in C6/36 cells using a pUb vector. At 16-h post-transfection, cell supernatant was replaced with EMEM at pH 7.4 or pH 6.5. At 1 h after replacement, cells were fixed with 4% paraformaldehyde for 15 min at room temperature and stained with 5 g/mL Hoechst 33342 in order to visualize nuclei. Arrowheads show multiple nuclei in a single cell, which is indicative of cell fusion. Scale bars, 50 m.

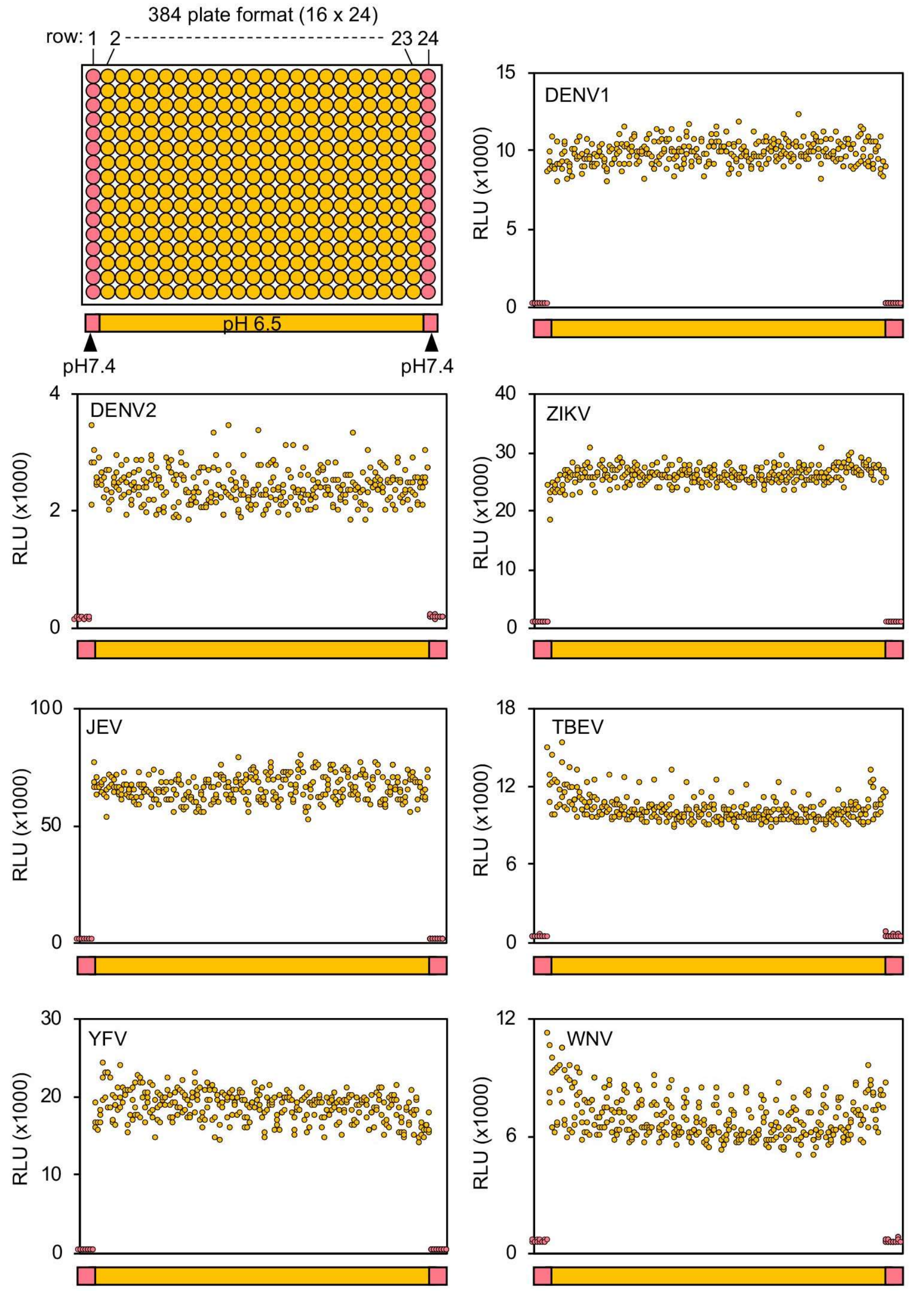

Figure S5 Validation of DSP-assay efficacy for HTS using flavivirus E proteins. The DSP assay was evaluated for its suitability as an HTS method using a 384-well format. Effector C6/36 cells expressing the E protein of each flavivirus and DSP1-7 and target cells expressing the same E protein as in effector cells and DSP8-11 were prepared and mixed as described in Materials and Methods. Mixed cells were incubated at pH 7.4 in 16 wells in the first row and in the final row (row 24) (pink wells). Other wells from rows 2 to 23 contained cells suspended in EMEM at pH 6.5 (yellow wells). Cells were incubated for 1 h at 28°C. Each dot represents RL activity shown in relative light units (RLUs). Signal/Background ratios and Z factors were calculated based on these results are shown in Table 1.

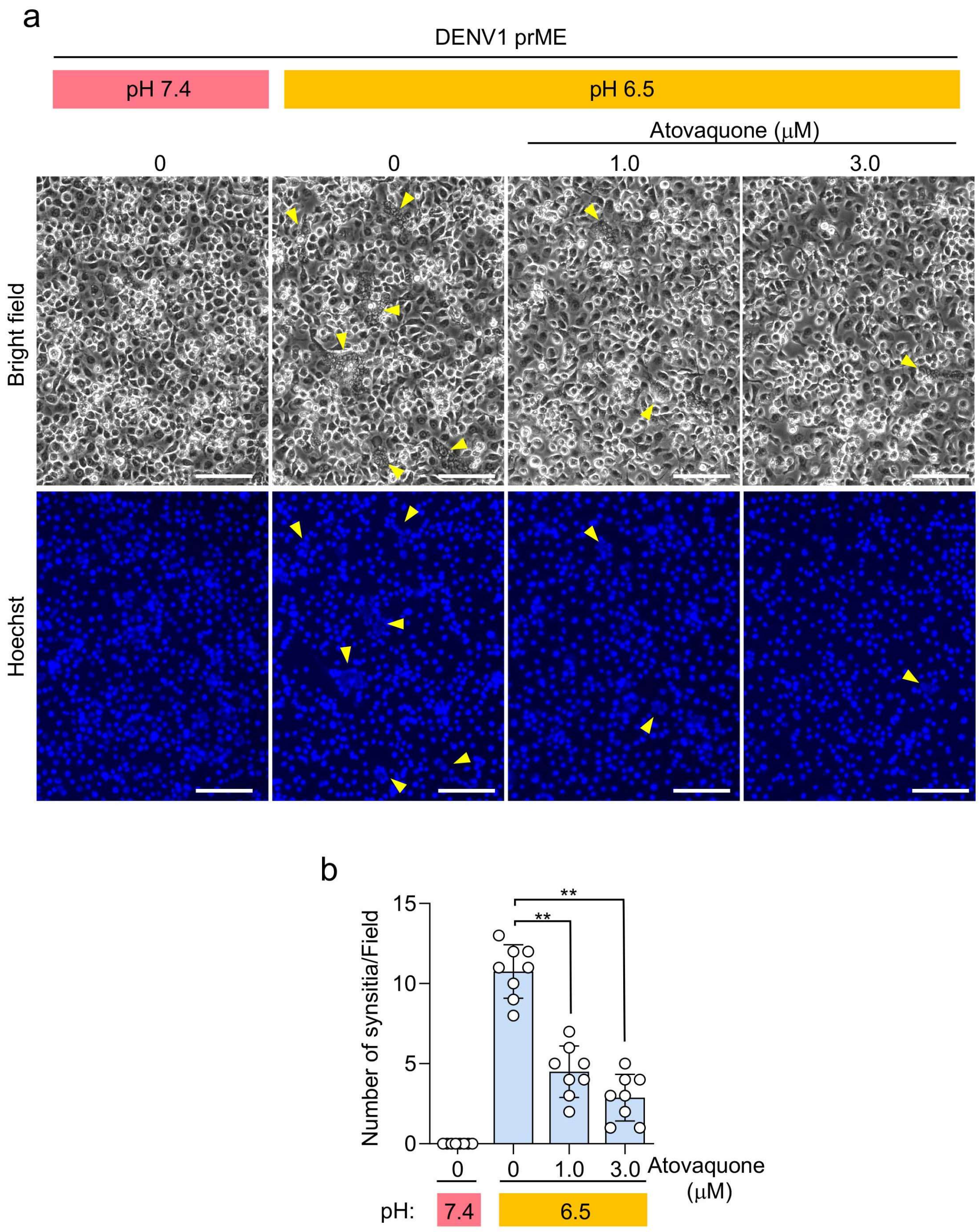

Figure S6 Inhibition of DENV1 E protein-mediated cell fusion by atovaquone. **a** DENV1 prME was transiently expressed in C6/36 cells using a pUb vector. At 16-h post-transfection, cell supernatant was replaced with EMEM at pH 7.4 without atovaquone or pH 6.5 with or without atovaquone. At 1 h after replacement, cells were fixed with 4% paraformaldehyde for 15 min at room temperature and stained with 5 g/mL Hoechst 33342 staining to visualize nuclei. Arrowheads show multiple nuclei in a single cell, which is indicative of cell fusion. **b** Fused cells per field in **a** was quantified. Statistical analysis was performed using Student's t test with Bonferroni correction. **$p < 0.01$. Scale bars, 50 m.

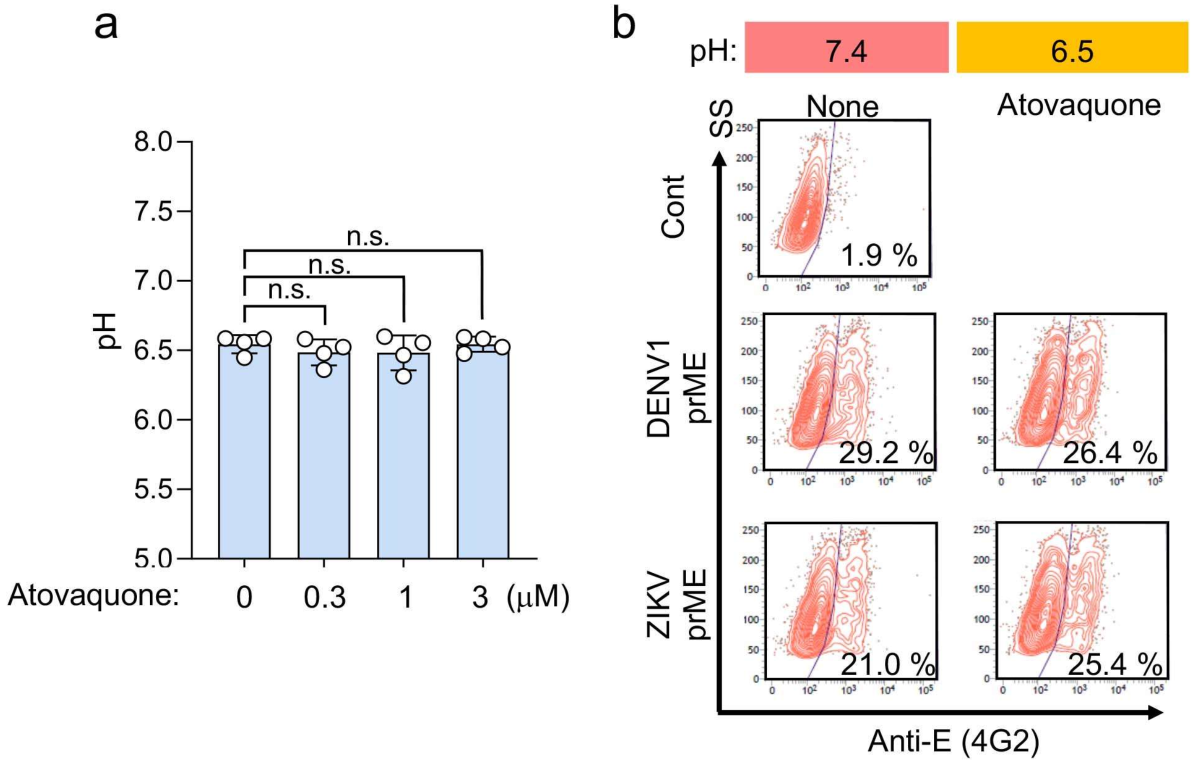

Figure S7 No alteration in medium pH or surface E protein expression levels by atovaquone treatment. **a** The effect of atovaquone treatment on medium pH. At 1 h after atovaquone treatment at low pH, pH values of cell supernatant were measured. **b** The effect of atovaquone treatment on surface E protein expression. DENV1 or ZIKV prME were transiently expressed in C6/36 cells using a pUb vector. At 16-h post-transfection, cell supernatant was replaced with EMEM at pH 7.4 or pH 6.5 with 3 M atovaquone. At 1 h after replacement, cells were fixed with 4% paraformaldehyde for 15 min at room temperature and stained with 4G2 to analyze surface expression of E protein without cell permeabilization. Statistical analysis was performed using Student's t test with Bonferroni correction. ns: no significant difference.
